# Inadequate intensive care physician supply in France: a point-prevalence prospective study

**DOI:** 10.1186/s13613-024-01298-y

**Published:** 2024-06-18

**Authors:** Sacha Sarfati, Stephan Ehrmann, Dominique Vodovar, Boris Jung, Nadia Aissaoui, Cédric Darreau, Wulfran Bougouin, Nicolas Deye, Hatem Kallel, Khaldoun Kuteifan, Charles-Edouard Luyt, Nicolas Terzi, Thierry Vanderlinden, Christophe Vinsonneau, Grégoire Muller, Christophe Guitton, CEMIR (Collège des Enseignants de Médecine Intensive Réanimation, French National College of Intensive Care Scholars), CNP MIR. (Conseil National Professionel de Médecine Intensive Réanimation, French National Council of Intensive Care Medicine)

**Affiliations:** 1 Medical Intensive Care Unit, Normandie Univ, UNIROUEN, UR 3830, CHU Rouen, 76000 Rouen, France; 2 Médecine Intensive Réanimation, INSERM CIC 1415, CRICS-TriggerSEP F-CRIN Research Network and Centre d’études Des Pathologies Respiratoires, INSERM U1100, Tours University, Tours, France; 3 Centre Antipoison de Paris, Hopital Fernand Widal, 75010 Paris, France; 4 Université Paris Cite, UFR de médecine, 75010 Paris, France; 5 Inserm UMR-S 1144 - Faculté de Pharmacie, 75006 Paris, France; 6 Médecine Intensive Réanimation, INSERM PhyMedExp, Université de Montpellier, CHU Montpellier, France; 7 Médecine Intensive Réanimation Hôpital Cochin, APHP, Paris, France; 8 Université Paris CIté, INSERM U 978, Équipe 4, AfterROSC, Paris, France; 9 Service de Réanimation Médico-Chirurgicale, CH Le Mans, Le Mans, France; 10 Paris Cardiovascular Research Center (PARCC), INSERM Unit 970, Paris, France; 11 Ramsay Générale de Santé, Hôpital Privé Jacques Cartier, Paris, France; 12 AfterROSC Network, Paris, France; 13 Medical & Toxicological Intensive Care Unit, UMR-S 942, Inserm, Lariboisiere University Hospital, APHP, Paris, France; 14 Intensive Care Unit, Cayenne General Hospital, Cayenne, French Guiana; 15 Tropical Biome and Immunopathology CNRS UMR-9017, Inserm U1019, Université de Guyane, Cayenne, French Guiana; 16 Service de Réanimation Médicale, GHRMSA, Hôpital Emile Muller, Mulhouse, France; 17 Médecine Intensive Réanimation, Institut de Cardiologie, Assistance Publique-Hôpitaux de Paris, Paris, France; 18 UMRS 1166, Sorbonne Université, GRC 30, RESPIRE, ICAN Institute of Cardiometabolism and Nutrition, Paris, France; 19 Medical Intensive Care Unit, University Hospital of Grenoble Alpes, Grenoble, France; 20 Medical Intensive Care Unit, University of Rennes, Rennes, France; 21 Médecine Intensive Réanimation, Groupement Hospitalier de L’Institut Catholique de Lille, FMMS - ETHICS EA 7446, Université Catholique de Lille, Lille, France; 22 Service de Médecine Intensive Réanimation, Centre Hospitalier de Béthune, Béthune, France; 23 CRICS_TRIGGERSep F-CRIN Research Network, Centre Hospitalier Universitaire (CHU) d’Orléans, Médecine Intensive Réanimation, Université de Tours, MR INSERM, 1327 ISCHEMIA, Université de Tours, 37000 Tours, France; 24 Faculté de Santé, Université d’Angers, Angers, France

**Keywords:** ICU, Intensivist, Medical staffing, Physician shortage

## Abstract

**Background:**

The COVID-19 pandemic has highlighted the importance of intensive care units (ICUs) and their organization in healthcare systems. However, ICU capacity and availability are ongoing concerns beyond the pandemic, particularly due to an aging population and increasing complexity of care. This study aimed to assess the current and future shortage of ICU physicians in France, ten years after a previous evaluation. A national e-survey was conducted among French ICUs in January 2022 to collect data on ICU characteristics, medical staffing, individual physician characteristics, and education and training capacities.

**Results:**

Among 290 ICUs contacted, 242 responded (response rate: 83%), representing 4943 ICU beds. The survey revealed an overall of 300 full time equivalent (FTE) ICU physician vacancies in the country. Nearly two-thirds of the participating ICUs reported at least one physician vacancy and 35% relied on traveling physicians to cover shifts. The ICUs most affected by physician vacancies were the ICUs of non-university affiliated public hospitals. The retirements expected in the next five years represented around 10% of the workforce. The median number of physicians per ICU was 7.0, corresponding to a ratio of 0.36 physician (FTE) per ICU bed. In addition, 27% of ICUs were at risk of critical dysfunction or closure due to vacancies and impending retirements.

**Conclusion:**

The findings highlight the urgent need to address the shortage of ICU physicians in France. Compared to a similar study conducted in 2012, the inadequacy between ICU physician supply and demand has increased, resulting in a higher number of vacancies. Our study suggests that, among others, increasing the number of ICM residents trained each year could be a crucial step in addressing this issue. Failure to take appropriate measures may lead to further closures of ICUs and increased risks to patients in this healthcare system.

## Background

In the recent COVID-19 pandemic outbreak, particular attention has been paid to intensive care units (ICUs) and their organization [1]. In many affected countries, bed capacity and availability of ICUs were reported daily in mainstream media. In the surge of the pandemic, healthcare authorities were faced with the fact that ICUs require large amounts of resources including a highly specialized workforce and equipment [2]. However, ICU capacity and availability are not only a pandemic issue. In the context of an aging population and the development of complex care among populations of increasing frailty, securing the availability of ICU beds has become a permanent issue. The shortage of ICU beds is a threat to patient safety both in hospital wards and emergency departments and the transfer of critically ill patients is associated with increased morbidity and mortality [3]. Therefore, maintaining an adequate capacity and distribution of ICUs nationwide is crucial to provide high-quality care in a modern healthcare system to meet the population’s needs.

Among the most important resources required to set up an ICU, the medical workforce is fundamental. In France, ICUs are staffed with board certified intensive care specialists (i.e., closed ICUs) [4], as the best evidence has shown the benefits of this type of organization [5–8]. According to French regulations, board certified intensivist must be available and onsite 24 h a day, 7 days a week [9]. However, the number of physicians required to establish and operate an ICU is not precisely regulated. The question of the target for ICU staffing has long been debated worldwide over the years of development of this specialty now crucial to modern health care systems [10]. Several intensive care medicine (ICM) societies have set up guidelines on the optimal number of physicians required in ICUs [11–13].

In France, there is no comprehensive up to date national administrative register of physicians working in ICM. In 2012, Annane et al. [14] conducted a study on the demographics and structures of French ICUs, which showed that the number of ICU physicians was much lower than required by guidelines and that more than three-quarters of ICUs had a medical team of fewer than seven physicians, impacting quality of care during night shifts. Since then, there has been no further evaluation of ICU physician supply at a national level. This evaluation is necessary in view of past concerns about ICU staffing in France and the future challenges associated with changes in ICM practice and the central role of ICUs in the healthcare system. An accurate assessment of the medical staffing of French ICUs, as well as current and future shortages was therefore mandatory to anticipate future healthcare needs.

We designed a point-prevalence prospective study via an e-survey sent to French ICUs in January 2022. The aim of this study was to determine the current state of physician supply in ICUs in France 10 years after the previous study [14]. Gathered information aimed at shedding light on the workforce to be trained in the coming years to meet the demand for intensive care across the country with adequate quality and safety of patient care.

## Methods

### Study design

We conducted a closed national (including overseas regions) prospective web-based e-survey, among heads of French stand-alone adult ICUs in the public and private sectors and in university-affiliated or non-university-affiliated hospitals, in January 2022.

### Development of the questionnaire

We divided the e-survey into three main parts. The first part aimed to collect structural, organizational, and activity data from the ICUs. Particular attention was paid to ICU capacity in terms of beds. In France, ICU beds are divided into two levels of care (LOC), corresponding to Level I and Levels II & III, as described by the European Society of Intensive Care Medicine Working Group on Quality Improvement [11]: LOC III represents patients with multiple acute vital organ failure of an immediate life-threatening character; LOC II represents patients requiring monitoring and support of only one acutely failing vital organ system. LOC I patients experience signs of organ dysfunction necessitating continuous monitoring and minor pharmacological or device-related support.

The second part aimed to collect aggregated data on ICU medical staffing, focusing on the number of vacancies, the teaching capacity of the unit for residents, and opportunities for growth in each ICU. The supply of medical staff was assessed by reporting the total number of physicians in persons and in full-time equivalents (FTE), considering the type of contract, such as academic staff who were counted as 0.5 FTE for clinical work as they are half time devoted to academic work.

The third section focused on the individual data of each physician working in these ICUs. This section included demographic characteristics (age and gender) as well as the type of position for each physician. In this section, we also sought to determine the proportion of physicians working in ICUs who were board certified either in anesthesiology and intensive care or in ICM. While most French ICU physicians are board certified in these fields, a number are recruited into ICUs without prior intensive care training due to the high demand for medical professionals in these units. Although they referred to individual items, these questions like the entire e-survey, were addressed to the heads of the ICUs.

Overall, the e-survey included 80 nonrandomized items without adaptive/alternate questions. For all the data collected, it was specified that temporary measures and organizational changes related to the COVID-19 pandemic should not be taken into account, in order to assess baseline ICU characteristics in the absence of any temporary measure.

### Definitions

Vacancies were defined as the number of currently vacant medical positions (FTE) budgeted and accepted by hospital management. We defined a travelling physician as a physician who temporarily works in an ICU to meet short-term staffing needs, regardless of his or her other medical activities. In our study, the term 'ICU physicians' encompasses any physician working in an ICU, regardless of their training or board-certified specialty. The terms ‘intensivist’ or ‘intensive care specialist’ are specifically used to denote physicians who have undergone training in intensive care.

### Recruitment process and description of the sample of ICUs with access to the e-survey

In France, health authorities provide health facilities with authorizations that are mandatory to open intensive care beds. From the listing of these administrative authorizations, we selected 290 French stand-alone adult ICUs. We excluded from the survey, pediatric ICUs (patients under 18 years of age) whose physicians are mainly trained in pediatrics. The study focused on closed ICUs where patient care is transferred to a physician belonging to the medical team dedicated to the ICU [4, 11]. Open ICUs were not included due to significant differences in their medical workforce configurations. This exclusion applied to specialized surgical ICUs, which share staff with larger anesthesiology departments, and stand-alone intermediate care units with only level I beds not associated with a level II/III ICU, ensuring homogeneity in the sample. To verify the conformity and updating of the list provided by the health authorities, the information was checked by the local and regional referents of the French National College of Intensive Care Scholars (*Collège des Enseignants de Médecine Intensive Réanimation*, CEMIR).

### Conduct of the study

The e-survey was hosted on the LimeSurvey (https://www.limesurvey.org/, version 5.2.9) platform. An email was sent in January 2022, to the heads of ICUs included in the study, proposing to participate in the e-survey through a specific link for each ICU to avoid the survey being completed twice by the same ICU. No incentives were offered. Heads of ICUs could complete the e-survey for 2 weeks from the time they received the invitation email. If there was no response after 2 weeks, they received reminder emails at the end of week 2 and week 4 and, again if there was no response, they were contacted directly by phone at the end of week 5 and week 6. The survey could be filled in over several days saving previous data input, and it was possible to modify the data already filled in before final validation. A completeness check of the answers filled in before submitting the questionnaire was implemented on the platform to maximize the number of completed items.

### Analysis of the surveys

The participation rate was defined as the ratio of the number of ICU heads who answered at least one question in the e-survey to the total number of ICU heads contacted. The completion rate was the ratio of the number of ICU heads who completed the survey in full to the total number of ICU heads contacted. Continuous and qualitative variables were expressed as median [interquartile range] and counts (percentage), respectively.

We focused here on the analysis of the results to assess the adequacy of the number of ICU physicians and the current capacity of French ICUs. Therefore, we analyzed the answers to the e-survey to present the overall characteristics of ICUs, the medical staffing of French ICUs, the individual medical staff characteristics, and finally the education and training capacities. In the medical staffing section, we set out to identify the staffing characteristics that could significantly alter the ability of an ICU medical team to provide appropriate care or to remain open. We defined significant shortage of medical staffing as (1) a medical staff of five or fewer physicians (FTE) or (2) a vacancy rate of more than 20% of the medical staff (FTE). We considered an ICU medical team as being in a situation of critical shortage if it met one of the above criteria and had at least one staff member expected to retire within 5 years. The analysis of medical staff characteristics was based on the answers from the individual data of each physician.

### Ethical, confidentiality and data process

The e-survey was conducted according to the Checklists for Reporting Results of Internet E-Surveys (CHERRIES) [15].

The survey was reviewed and approved by the board of the CEMIR. The review board of the Hospital of Le Mans (France) approved the study protocol (REF-0020-Enquête CEMIR). On the first page of the e-survey, participants were informed about the length of time allocated to complete the survey, that the investigators were members of the CEMIR board, and about data storage. The data from e-surveys were stored under the supervision of the data controller at the Hospital of Le Mans, France, in accordance with French regulations.

## Results

**Fig. 1:**
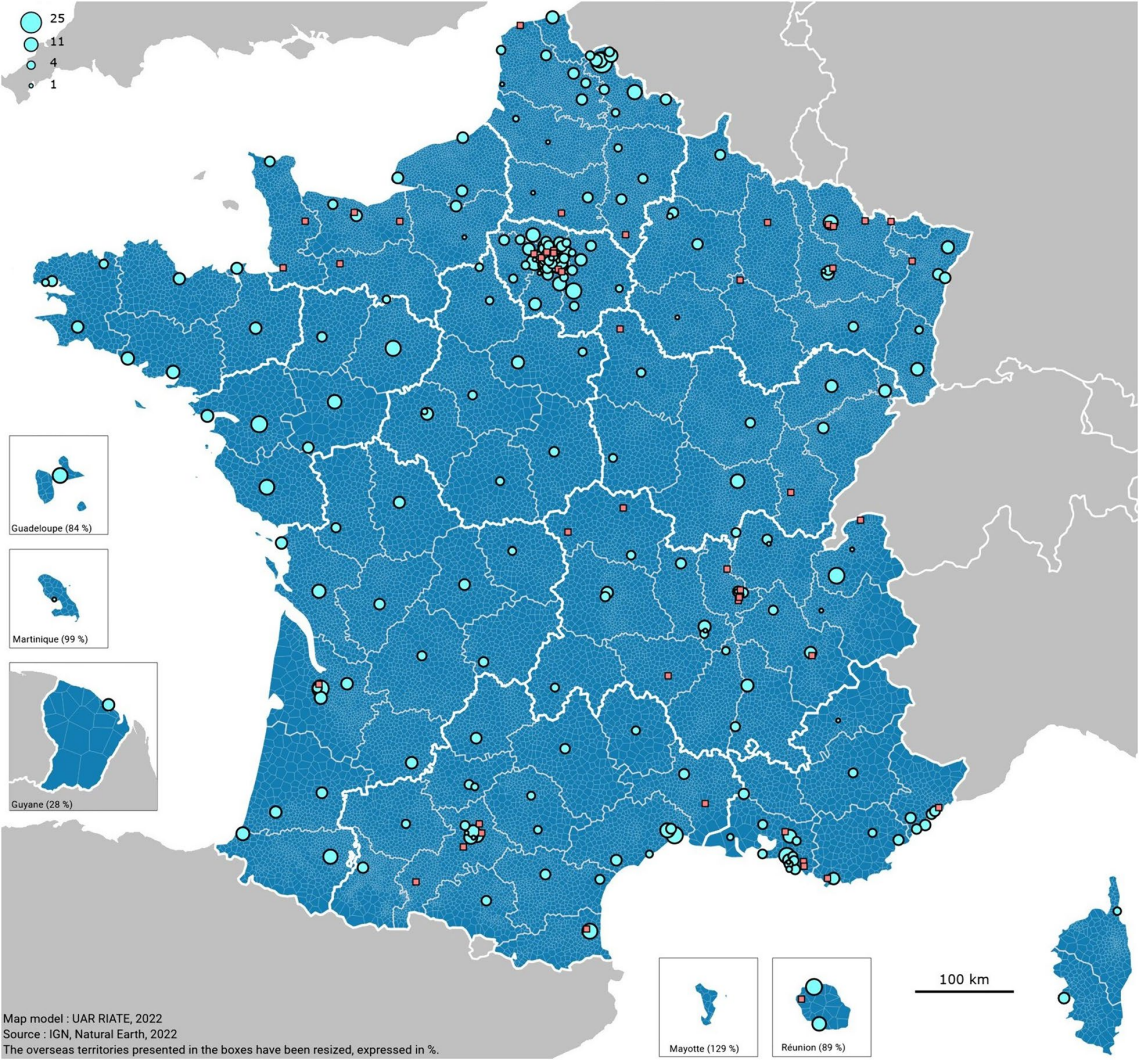
Geographical location of the 290 ICUs contacted. Light blue circles represent responding ICUs, with the size of the circle indicating the number of physicians (full time equivalent). The responding ICUs that did not answer this question of the survey were assigned a circle of size 1. The orange squares represent the non-responding ICUs

Of the 290 ICUs contacted, 242 answered at least one question (response rate: 83%) and 218 completed the survey entirely (completion rate: 75%). The geographical location of the responding and non-responding ICUs is presented in Fig. 1.

### ICU characteristics

Responding ICUs were in hospitals with a median capacity of 450 [304–726] beds (Table 1). More than eight out of ten (84%, n = 204) ICUs admitted both medical and surgical patients. In 79% (n = 192) of cases they were in public hospitals and the most common location was in non-university public hospitals (72% of public and 57% of all ICUs, n = 138). A total of 4,943 ICU beds were reported nationally. Of these, only 94% were open, representing 1572 level I beds (34%) and 3,059 level II and III beds (66%). The median number of open beds per ICU was 18 [14–24] (12 [9–15] level II/III and 6 [4–8] level I beds). Almost one-third of ICUs (32%, n = 73) reported at least one closed bed. These beds were closed in more than two-thirds of cases (71%, n = 223) because of nursing vacancies and in 40% of cases because of physician vacancies (n = 125), both could occur at the same time. In 2021, 137,822 patients were admitted to responding ICUs, with a median number of admissions per ICU of 556 [386–760] and a median length of stay of 7.9 days [6.23–9.5]. The median simplified acute physiology score II (SAPS II) [16] reported for level II/III beds was 44 [40–48].

**Table 1:** Characteristics of responding ICUs, categorized by hospital type, ICU type, hospital and ICU capacity, and patient characteristics. Categorical variables are presented as n (%) and continuous variables as median [IQR]

Characteristics of responding ICUs characteristics		Number of respondents (N)
Total ICUs contacted	290	
Participation rate	242 (83.4)	
Completion rate	218 (75.1)	
Hospital type		
Public	192 (79.3)	242
University	54 (28.1)	242
Non-university	138 (71.9)	242
Private	44 (18.2)	242
For-profit	29 (65.9)	242
Non-profit	15 (34.1)	242
Military	6 (2.5)	242
ICU type		
Medical only	31 (12.8)	242
Surgical only	2 (0.8)	242
Mixed	204 (84.3)	242
Other	5 (2.1)	242
Hospital & ICU capacity		
Hospital beds	450 [304–726]	228
Total ICU beds supplied	4943	232
Total ICU beds opened	4631(93.6)	232
Level of care II & III	3059 (66.0)	232
Level of care I	1572 (34.0)	232
Open beds per ICU	18 [14–24]	232
Level of care II & III	12 [9–15]	232
Level of care I	6 [4–8]	232
Total ICU beds closed	315 (6.4)	232
ICUs with at least 1 closed bed	73 (31.5)	232
ICU beds closed for medical staff vacancy	125 (39.7)	229
ICU beds closed for nursing staff vacancy	223 (70.8)	231
Patient characteristics (Level of care II & III)		
Mean SAPS II score in 2021	44 [40–48]	224
Mean length of stay (days) in 2021	7.9 [6.23–9.5]	227
Total number of admissions in 2021	137822	226
Number of admissions in 2021	556 [386–760]	226

The number of respondents for each item corresponds to the number of responses collected in the e-survey. Level of care (LOC) is defined according to the European Society of Intensive Care Medicine Working Group on Quality Improvement [11]: LOC III represents patients with multiple acute vital organ failure of an immediate life-threatening character; LOC II represents patients requiring monitoring and support of only one acutely failing vital organ system. LOC I patients experience signs of organ dysfunction necessitating continuous monitoring and minor pharmacological or device-related support. Patient characteristics are reported based on 2021 statistics for LOC II & III. Mean SAPSII and length of stay are calculated per ICU

*ICU* intensive care unit; *IQR* inter-quartile range; *n* number; *SAPS* simplified acute physiology score II

### Medical staffing of French ICUs

Responding ICUs had 1862 FTE physician positions (Table 2). However, only 1,562 (84%) FTE physician positions were filled, corresponding to 1,780 practitioners. The number of doctors per ICU was 7.0 [5.0–10.0]. This corresponded to 6.5 [5.0–8.25] FTE doctors per ICU. The number of doctors (FTE) per ICU bed was 0.36 [0.29–0.47]. Among these doctors, 57 (3%) were expected to retire within one year and 184 (10%) within five years. The number of 24-h shifts per doctor was 5.0 [4.0–5.0] per month.

**Table 2:** Medical staff characteristics for responding ICUs with a focus on medical vacancies and medical teams with a critical shortage

Medical staffing characteristics		Number of respondents (N)
Total physicians	1780	223
Physicians per ICU	7.0 [5.0–10.0]	223
Total physicians (FTE)	1562.15	223
Physicians (FTE) per ICU	6.5 [5.0–8.25]	223
Physicians (FTE) per ICU bed	0.36 [0.29–0.47]	223
Monthly 24-h shift/physician	5 [4, 5]	234
Total one-year projected retirement	57 (3.2)	224
Total five-year projected retirement	184 (10.3)	224
Medical vacancies		
Total physician vacancies (FTE)	299.75 (16.1)	223
In public non-university hospitals	247.40 (82.5)	223
ICUs with at least one physician vacancy	140 (62.8)	223
In public non-university hospitals	108 (77.1)	223
ICUs with more than one physician vacancies (FTE)	100 (44.8)	223
In public non-university hospitals	82 (82.0)	223
ICUs with a need for a travelling physician within the past year	77 (34.4)	224
ICU medical teams in critical shortages		
(1) ICUs with ≤ 5 physicians (FTE)	65 (29.1)	223
(2) ICUs with a vacancy rate of > 20% of physicians (FTE)	88 (39.5)	223
ICUs with (1) OR (2)	108 (48.4)	223
ICUs with [(1) OR (2)] AND ≥ 1 FTE projected retirement within five years	60 (26.9)	223

Categorical variables are presented as n (%) and continuous variables as median [IQR]. The number of respondents for each item corresponds to the number of responses collected in the e-survey. We defined significant medical shortage as (1) a medical staff of five or fewer physicians (FTE) or (2) a vacancy rate of more than 20% of the medical staff (FTE). We considered an ICU medical team as being in a situation of critical shortage if it met one of the above criteria and had at least one staff member expected to retire within 5 years

*FTE* full-time equivalent; *ICU* intensive care unit; *IQR* inter-quartile range; *n* number

With a total of 300 FTE vacancies, nearly two-thirds of the participating ICUs (63%, n = 140) reported at least one physician vacancy and almost one-half (45%, n = 100) reported more than one physician vacancy. Consistently, more than one-third of ICUs (35%, n = 77) had to rely on a travelling physician within the previous year. The ICUs most affected by physician vacancies were the ICUs of public non-university hospitals which represented 77% of ICUs with at least one physician vacancy and 82% of ICUs with more than one physician vacancy.

Concerning critical shortages, 29% (n = 65) of participating ICUs had 5 or fewer physicians (FTE), placing a very high pressure on 24/7 physician availability, and 40% (n = 88) had a physician (FTE) vacancy rate greater than 20%. In total, almost half (48%) of responding ICUs met at least one of these criteria. Among them, 60 ICUs (27% of all ICUs) also had one or more FTE position retiring within five years and were therefore in a situation of critical shortage.

### Medical staff characteristics

**Fig. 2:**
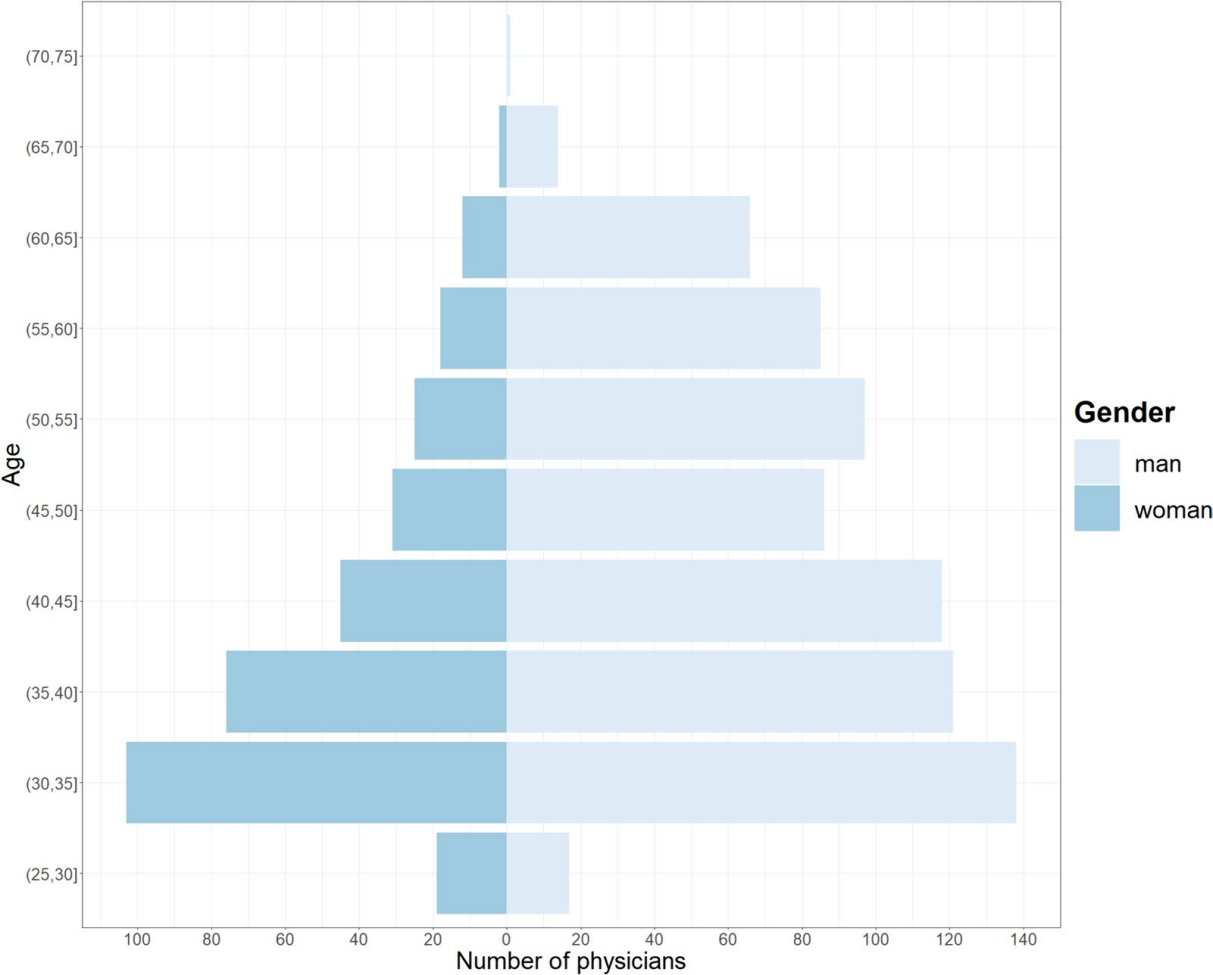
Number of physicians working in French intensive care units by 5-year age group and by gender

Of the 1,780 ICU physicians identified in this study, we collected individual data on 1,643 of them. The physicians working in the responding ICUs were men in more than two-thirds of cases (70%, n = 1139) and had a median age of 42 years [35–52] (Fig. 2, Table 3). Almost one-third of them (30%) were over 50 years old, and 9% were over 60 years old. The gender ratio of physicians, although unbalanced in favor of men overall, tended to be more balanced in the younger age groups (Fig. 2). More than two-thirds of ICU physicians had a permanent position (78%, n = 1,254) and a full-time position (78%, n = 1,265). Regarding the medical qualifications of ICU physicians, 15% (n = 248) were not board certified in intensive care, and 9% (n = 142) had a foreign medical qualification.

**Table 3:** Medical staff characteristics of responding ICUs grouped by demographics, type of position and type of medical qualification

Medical staff characteristics		Number of responses (N)
Demographics		
Age of physicians	42 [35–52]	1080
> 50 y, n (%)	324 (30.0)	1080
> 60 y, n (%)	97 (9.0)	1080
Gender of physicians, man	1139 (69.6)	1637
Type of position		
Permanent position	1254 (77.7)	1615
Full-time position	1265 (78.4)	1615
Academic position	176 (10.9)	1615
Type of medical qualification		
Not board certified in intensive care	248 (15.1)	1643
Foreign medical qualification	142 (8.9)	1597

Categorical variables are presented as n (%) and continuous variables as median [IQR]. The number of responses for each item corresponds to the number of responses for individual staff member data collected in the e-survey

*IQR* inter-quartile range; *n* number

### Education and training capacities

Among ICUs that responded to the survey, 85% (n = 190) provided training for residents (all specialities combined) representing 1062 residents (Table 4). The median number of residents per physician (FTE) was 0.63 [0.24–0.95]. ICU heads were asked to simulate the maximum number of resident positions. Nationally, the maximum resident capacity was reported to be 1374, representing an increase of 312 residents compared to current staffing levels, thus more residents could easily be trained. These additional resident positions were distributed among 71% of all responding ICUs.

**Table 4:** Education and training capacities of responding ICUs

Education and training		Number of respondents (N)
Total residents trained in ICUs	1062	224
ICUs with residents	190 (84.8)	224
Resident/Physician (FTE) ratio	0.63 [0.24–0.95]	223
Total maximum resident capacity	1374	218
ICUs with additional resident positions	154 (70.6)	218

Categorical variables are presented as n (%) and continuous variables as median [IQR]. The number of respondents for each item corresponds to the number of responses collected in the e-survey

*FTE* full-time equivalent; *ICU* intensive care unit; *IQR* inter-quartile range; *n* number

## Discussion

Our study is the only recent assessment of the supply and shortage of ICU physicians at the level of an entire country. Among 290 ICUs identified in France, 242 responded to our survey, representing 4943 ICU beds and 1780 physicians. Hence, the beds we surveyed represent 70% of level II and III ICU beds in France and the level I beds directly associated with them.

The main result of our study is the important shortage of ICU physicians. There were 140 ICUs with at least one physician vacancy (63% of all ICUs) for an overall of 300 FTE physician vacancies in the country**.** The majority of affected ICUs were those in public non-university hospitals, which accounted for 77% of ICUs with at least one physician vacancy and 82% of ICUs with more than one physician vacancy. In addition, the retirements planned for the next five years represent around 10% of the workforce. Finally, we identified 60 ICUs experiencing a critical shortage of ICU physicians.

Compared to a similar study conducted in 2012 [14], we observed an increase in the inadequacy between ICU physician supply and demand, in line with the increased number of patients admitted to ICUs, and a very small increase in the number of ICU beds. These studies are broadly comparable in terms of methodology, type and number of ICUs surveyed, and response rate. In 2012, 290 ICUs were identified and showed a response rate of 74.1% (215 responding ICUs). In 2012, the median number of admissions per ICU was 445, increasing by 25% over ten years to 556 in 2022. The mean length of stay and mean SAPSII were similar in the two studies. In their study, authors reported 1331 ICU physicians (1164.5 FTE) and 121 vacancies in 2012. Compared to the present study, this represents a 148% increase in vacancies for ICU physicians over ten years. Between 2012 and 2022, the increase in supply of ICU physicians, the median number of physicians per ICU (FTE) increasing from 5.0 to 6.5 over the period, was insufficient to meet the demand for ICU physicians. As a result, the number of ICUs with at least one vacant physician position increased by 77%, particularly in public non-university hospitals, where the increase reached 112% between the two studies.

In France in 2022, most of the responding ICUs operated with a small number of physicians. Half of the ICU medical teams in France consisted of fewer than 7 physicians. This corresponds to a ratio of 0.38 FTE / ICU beds. This low number of physicians per team and per ICU bed raises questions about safety and quality of care. There is evidence that high-intensity medical staffing reduces hospital and ICU mortality [6] and increases quality indicators of medical management in ICUs [17]. Therefore, appropriate medical staffing of ICUs is of great importance for the quality of ICU care. In addition, evidence of the harmfulness of night and extended work hours to health practitioners and patient safety, as well as staff workload is now well documented [18–20]. The frequency of night shifts per physician has also been shown to have an impact on burnout among intensivists [21]. Our study found a median of five night shifts per month per physician, i.e., more than one per week. As a consequence of such small teams, 34.4% of ICUs reported having to rely in part on travelling physicians temporarily hired to cover shifts. Although this is currently necessary, in the closed-ICU model, associated with better outcomes, medical teams should be self-sufficient in terms of staffing. There is currently no legal ratio of physicians per patient in ICU in France, but scientific societies have sought to establish a minimum threshold of 0.60 to 0.80 FTE intensivists per bed for good patient care [11, 12, 22, 23]. The scarcity of ICU physicians in France becomes even more pronounced when comparing the physician-to-ICU-bed ratio with these internationally recommended standards. These recommendations are based on evidence that medical team size is an important factor in ensuring quality of care, training future physicians and maintaining a stable ICU workforce, taking into account all aspects of physicians’ work, including teaching, meeting with patients’ families and relatives, procedures, consultations, non-ICU duties and administration. Intensive care capacity must therefore be considered not only in the light of the current shortage of physicians but also in the perspective of a need to increase the size of medical teams if we are to maintain adequate quality and safety of care.

The risk of a persistent shortage of ICU physicians in an environment where the teams are already small and under pressure is the closure of ICUs or intensive care beds in the short to medium term as part of the workforce retires. The impact of this ongoing phenomenon is already obvious, with respondents to our survey reporting a total of 125 beds closed due to medical staff vacancies. In addition, we identified 60 ICUs as being in a situation of critical shortage due to vacancies and impending retirements (5 or fewer FTE physicians or a vacancy rate of above 20%, and one or more FTE positions retiring within five years). We chose these criteria as a team of six physicians would lead to a mean of more than five night shifts per month which has been shown to be associated with a high level of burn-out [20]. We considered the association of one of these criteria with one or more FTE positions retiring within five years as being in a situation of critical shortage. As a result, close to one-third of French ICUs are at risk of potential closure, posing a threat to the healthcare system.

The shortage of ICU beds and the high ICU capacity strain, even locally and temporarily, expose the population to significant risk [24–28]. The COVID pandemic was the most prominent example, but epidemic, environmental or accidental risks are ever-present at local and international levels and can quickly lead to a shortage of ICU beds. It has been shown that transfer of patients or delayed admission to ICU due to a lack of available ICU beds may impact outcome [3, 29, 30]. In addition, transfers require medical and paramedical staff, the resources of which are limited [31]. Therefore, ensuring local ICU bed availability is crucial for patient safety. In addition, the rapid progress of curative treatments in many diseases has led to the admission of older patients with more co-morbidities to intensive care, for their benefit [32, 33], and not at greater costs [34]. Today, ICUs have taken a central place in the healthcare system as an essential support for the implementation of new medical or interventional techniques. Maintaining access to intensive care for the entire population is therefore essential for safety and quality of care.

In the absence of an increase in the supply of ICU physicians through increased training, France is facing a permanent shortage and closure of ICU beds, with consequent risks to the population.

In their study, Annane et al. [14] advocated a significant increase in the number of new intensivists trained each year, which so far has not happened. As a result, ICU physician vacancies have increased, a significant proportion of physicians working in ICUs are not board-certified in ICM, and many foreign physicians have had to be recruited. Only in 2017 was the training of ICM specialists regulated with only a slow quantitative increase over the years to reach only 101 residents entering training in 2022. On average between 2022 and 2027, 80 new ICM specialists will graduate each year. This workforce will be complemented by physicians trained in anesthesiology & intensive care, who are much more numerous. However, despite their high level of qualification, only a small proportion of them will be working in ICM in the long term [35]. Based on the planned retirements, vacancies, and part-time employment rates identified in the present study, as well as the planned increase in ICU beds, the number of newly trained intensivists should at least double to address the ongoing shortage. Given that training capacity is currently underexploited with the potential to provide practical training for at least an additional 300 residents for each rotation in French ICUs, the shortage could be addressed by increasing the quotas set by the French health authorities of residents trained each year in ICM. Overall, without a very steep increase in these quotas, the shortage of ICU physicians will increase, since the number of newly trained ICM specialists is insufficient to meet the demand for intensivists reported in our study.

Although the data presented are specific to France, they are likely to provide insights for countries with similar healthcare contexts. In 2007, an international study reported comparable patient-to-intensivist ratios [36]. However, it is worth noting that at the time, many countries did not require the presence of an intensive care specialist on site 24 h a day, 7 days a week, nor did they require ICU physicians to be board certified in intensive care as is now the case in France. Internationally, studies have shown that a shortage of intensivists correlates with poorer patient outcomes in ICUs [6–8, 21, 26]. In response to a similar crisis 25 years ago [37], the United States implemented new ICU staffing standards, driven in part by the intensivist community, which have significantly improved the quality of care [38]. This requires a clear understanding of the existing staffing situation. Consequently, we encourage other countries to thoroughly evaluate their ICU physician workforce in order to avoid neglecting a potentially harmful shortage.

The main limitation of our study is the declarative aspect of the data. The e-survey was sent to the heads of the listed ICUs and therefore the accuracy of the data relies on the sincerity and knowledge of the respondents. This limitation is particularly important for the third part of the e-survey, which focuses on the individual data of each physician working in these ICUs. Indeed, data such as age or qualification may be inaccurately reported by the heads of ICUs. A second limitation of the present work is the point-prevalence method of the study, which does not allow us to present trends in our results. The repetition of this study in the future would be of great interest to follow changes in the supply of ICU physicians. Finally, although we insisted that responses should be given excluding possible measures specific to the COVID-19 crisis, it is likely that it was difficult to completely exclude this effect from our results.

## Conclusion

The French healthcare system is experiencing a shortage of critical care physicians. Our study shows a shortfall of 300 full-time equivalent (FTE) ICU physician. Moreover, it is projected that a further 171 FTE will retire within the next five years. In addition, French ICUs already exhibit small medical teams at risk of forthcoming closures. Our findings also show that the existing shortage is contributing to increased workloads and has led to the closure of some ICU beds. Given the essential role of ICUs in the wider healthcare system, these developments could potentially affect several other medical services. Our study suggests that, among others, increasing the number of ICM residents trained each year could be a crucial step in addressing this issue and preventing future ICU closures.

## Data Availability

The datasets used and/or analysed during the current study are available from the corresponding author on reasonable request.

## Abbreviations

CEMIR: *Collège des Enseignants de Médecine Intensive Réanimation*, French National College of Intensive Care Scholars
FTE: Full time equivalent
ICM: Intensive care medicine
ICU: Intensive care unit
LOC: Level of care
SAPS II: Simplified acute physiology score II
